# Export of ice-nucleating particles from watersheds: results from the Amazon and Tocantins river plumes

**DOI:** 10.1098/rsos.220878

**Published:** 2023-02-08

**Authors:** Annika Einbock, Emma Burtscher, Claudia Frey, Franz Conen

**Affiliations:** Department of Environmental Sciences, University of Basel, Bernoullistrasse 30, 4056 Basel Switzerland

**Keywords:** ice-nucleating particles, river plume, Amazon, Tocantins, watershed, atmosphere

## Abstract

We examined ice-nucleating particles (INPs) in the plumes of the Tocantins and Amazon rivers, which drain watersheds with different proportions of degraded land. The concentration of INPs active at −15°C (INP_−15_) was an order of magnitude lower in the Tocantins (mean = 13.2 ml^−1^; s.d. = 7.8 ml^−1^), draining the more degraded watershed, compared with the Amazon (mean = 175.8 ml^−1^; s.d. = 11.2 ml^−1^), where the concentration was also significantly higher than in Atlantic surface waters (mean = 3.2 ml^−1^; s.d. = 2.3 ml^−1^). Differences in heat tolerance suggest that INPs emitted by the Amazon rainforest to the atmosphere or washed into the river might originate from contrasting sources on top of and below the rainforest canopy, respectively. For the Amazon River, we estimate a daily discharge of 10^18^ INP_−15_ to Atlantic waters. Rivers in cooler climate zones tend to have much higher concentrations of INPs and could, despite a smaller water volume discharged, transfer even larger absolute numbers of INP_−15_ to shelf waters than does the Amazon. To what extent these terrestrial INPs become aerosolized by breaking waves and bubble-bursting remains an open question.

## 1. Introduction

Ice-nucleating particles (INPs) are a prerequisite for initial ice formation in clouds at temperatures warmer than −38°C [1,2]. Ice is the starting point for most precipitation above continents [3]. Land and ocean both emit INPs, but ice-nucleation active site densities in sea-surface aerosols are a few orders of magnitude lower than in continental boundary layer aerosols [4]. Some rivers have been reported to carry high concentrations of INPs that have been produced on land rather than in the river itself [5–9]. When discharged to the ocean, they probably enhance INP concentration at the surface of shelf waters, possibly creating source areas from where INPs of marine and terrestrial origins are aerosolized in parallel by wave-breaking and bubble-bursting. Earlier work on the number of biological INPs produced by decaying leaves has suggested that biological INP production on land may increase by orders of magnitude successively from tropical to temperate to cold climate zones [10,11]. Further, different INP densities are observed on contrasting types of vegetation, where land-use change is suspected to modify INP sources and thereby the formation of precipitation [12].

Perhaps, the study of INPs in rivers draining watersheds with different land cover can reveal effects of land-use change on INP production and, subsequently, on the atmospheric hydrological cycle [12,13]. Here, we report on INPs in the plumes of the Amazon and Tocantins rivers. The Amazon River drains the largest watershed worldwide and has an annual average flow rate at the Óbidos station of 150 500 m^3^ s^−1^ [14]. The watershed of the Tocantins River is about six times smaller and its average discharge at the Tucurui station is about 13 times smaller [15] than the Amazon. Seasonal changes in rainfall [15] are reflected in discharge, which in the Amazon ranges from approximately 100 200 to 240 000 m^3^ s^−1^ [14].

A much larger fraction of the Tocantins watershed has deteriorated due to deforestation and drought over the past decades than is the case for the Amazon watershed [16]. Unlike the Amazon, the Tocantins watershed also includes dryer climate types. Thus, effects of land-use change cannot be separated from climate effects. However, both climate change and deforestation are expected to lead to drier conditions in the Amazon region [17]. Therefore, INPs in the Tocantins watershed today might be a proxy for what they might become in the Amazon watershed in future. We expected differences in land cover between both watersheds to result in different INP populations in the plumes of the Amazon and Tocantins rivers. These differences should emerge primarily in INPs active at −15°C or above (INP_−15_), because most INP_−15_ are of biological origin and are ultimately derived from plants and associated microorganisms [18–20].

## 2. Material and methods

### 2.1. Land cover in the Amazon and Tocantins watersheds

**Figure 1.**
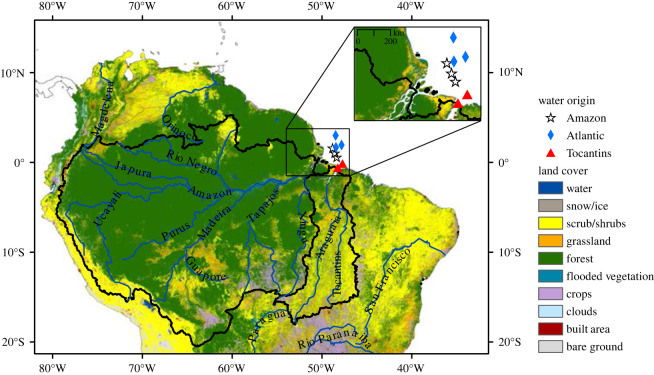
Land cover (100 × 100 m resolution) in the watersheds of the Amazon River (large, western area bordered by black line) and the Tocantins River (smaller, eastern area bordered by black line). Sampling locations in the Amazon (white stars) and Tocantins (red triangles) river plumes, and in the Atlantic (blue diamonds). Sources: Land cover: Sentinel-2, 2020, resampled at 100 m resolution (https://www.arcgis.com/apps/instant/media/index.html?appid=fc92d38533d440078f17678ebc20e8e2); watersheds: Agência Nacional de Águas e Saneamento Básico (ANA) (https://metadados.snirh.gov.br/geonetwork/srv/search?keyword=Ottobacia).

Currently, the majority of the Amazon River watershed is covered by forest (81%), followed by scrub and shrubs (10%) (figure 1). Grassland (4%) and crops (3%) cover a smaller proportion than in the Tocantins River watershed (9% and 7%, respectively), where the share of forest is only 36%, and scrub and shrubs are more abundant (45%) (figure 1).

### 2.2. Field sampling

Water was sampled towards the end of the rainy season from the plumes of the Amazon River and the Tocantins River during the cruise M174 of the research vessel R/V *Meteor* [21]. The focus of the cruise was on exploring the fate of nitrogen in the Amazon River plume and the western tropical North Atlantic [22]. Surface water pumped from the moon pool, an opening in the base of the hull 5.70 m below the water line, was collected in sterile polypropylene tubes (40 ml). To prevent eventual contamination with residues in the pumping system, the water was kept running for several minutes beforehand. Samples were immediately put into a −20°C freezer and remained frozen until analysis. High-resolution data on water quality was collected with the CTD-system ‘SBE 911plus’ (SN-0603, SEABIRD-ELECTRONICS, USA), with added sensors to measure pressure, temperature (2× SBE 3), conductivity (2× SBE 4), oxygen concentration (2× SBE 43), chlorophyll-a fluorescence (683 nm), turbidity, PAR, SPAR and nitrate (SUNA sensor).

Salinity data confirms that samples collected at the mouths of the Amazon and Tocantins rivers were indeed river water with little or no seawater (table 1). The fluorescence values were practically the same in both river plumes. Turbidity was lower by a factor of two or three in one sample from the Amazon River and one from the Tocantins River plume. Both of these samples also had a slightly enhanced salinity.

**Table 1.** Origin of samples analysed for their content of INPs and properties of the sampled waters.

origin of water	sample no.	date	time (UTC)	latitude	longitude	conductivity (mS cm^−1^)	salinity (PSU)	fluorescence (mg m^−3^)	turbidity (NTU)
Tocantins	1	23.04.2021	07:23	00°07.30' S	047°43.87' W	7.5	3.9	1.1	5.3
	2	23.04.2021	15:54	00°35.85' S	048°14.63' W	2.0	0.9	1.1	9.7
Amazon	3	24.04.2021	12:57	00°35.83' N	048°22.40' W	1.1	0.5	1.0	9.7
	4	24.04.2021	16:07	01°02.91' N	048°35.18' W	1.6	0.7	1.1	9.7
	5	24.04.2021	00:48	01°36.73' N	048°50.29' W	6.4	3.2	1.2	3.2
Atlantic	6	29.04.2021	07:40	03°00.15' N	048°28.91' W	57.3	35.1	0.09	0.08
	7	30.04.2021	16:09	04°24.35' N	049°07.29' W	57.4	36.0	0.22	0.08
	8	03.05.2021	20:00	05°02.01' N	050°21.12' W	57.5	35.6	0.08	0.08

### 2.3. Analysis of INPs

Immediately after melting a sample at room temperature, we transferred a subsample of 100 µl in each of 52 Eppendorf safe-lock tubes (0.5 ml) for analysis on the ice-nucleation detection apparatus described in Stopelli *et al*. [23]. Tubes were cooled at a rate of 0.3°C min^−1^ from 0 to −20°C. The freezing temperature of the subsample in each tube was detected automatically and the cumulative INP concentration at every 0.5°C step was calculated according to Vali [24]. When all 52 droplets had frozen before reaching −12°C, we reanalysed samples in a 1:10 dilution with ultrapure water (Sigma-Aldrich, W4502-1L) to extend the measurement range to −15°C. Duplicate blank tests with only ultrapure water confirmed it was free of INP_−15_. For characterizing INPs in terms of their heat sensitivity, we performed the same freezing assay two more times: after treating the same array of subsamples in their Eppendorf tubes for 10 min in a water bath at 60°C and after a second such treatment at 95°C. In the Atlantic water samples, we shifted measured values by 2°C towards the warmer end of the temperature scale to account for freezing depression due to high salt concentration. We derived the 2°C value from the difference between freezing spectra with Snomax in pure water and with Snomax in pure water to which we added an equivalent of 35 g l^−1^ NaCl.

## 3. Results and discussion

Cruise M174 gave us the opportunity to take a snapshot of INPs in a region we otherwise could not have accessed. The sampled river plumes integrate diversity and variation of innumerous watercourses that supply the Tocantins and Amazon rivers before they reach the Atlantic. Successive integration of distant tributaries with asynchronous variation within a large watershed confers a relative stability in terms of biotic and abiotic properties to the water discharged to the ocean [25]. Therefore, we expect our investigation to afford meaningful interpretation despite its snapshot character and the small number of samples.

### 3.1. Concentration of INPs in river plumes

**Figure 2.**
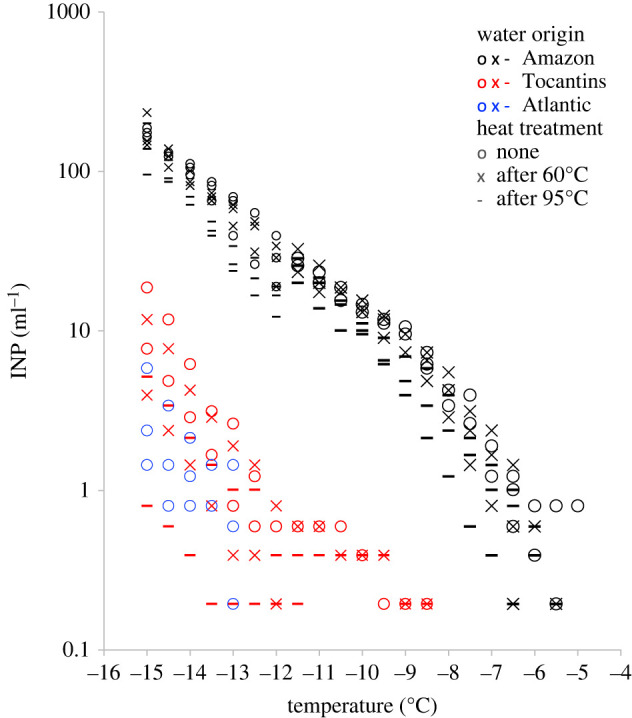
Concentration of INPs active between −5 and −15°C in three samples of the Amazon River plume (black), two samples of the Tocantins River plume (red) and three samples of Atlantic surface water (blue). The Atlantic samples, all containing more than 35 g salt l^−1^, were adjusted for freezing point depression. We analysed the same sample material three times: first, without any treatment (circles); second, after heating to 60°C (cross symbols); and third, after heating to 95°C (dash symbols). In addition, we analysed Amazon River plume samples in a 10-fold dilution with pure water (smaller symbols) to determine INP concentration at lower temperatures than was possible with undiluted samples (data in electronic supplementary material).

The onset of freezing in 1 ml of water was observed at −6.5°C in the Amazon River plume and at −12.5°C in the Tocantins River plume. Differences between samples taken from within the same river plume were small (figure 2). The number of INP_−15_ in 1 ml of water was one order of magnitude higher in the Amazon River plume (mean = 175.8; s.d. = 11.2) than the Tocantins River plume (mean = 13.2; s.d. = 7.8). While this difference was statistically significant (*p* < 0.001), the difference between the Tocantins River plume and Atlantic surface water (mean = 3.2; s.d. = 2.3) was much smaller and statistically insignificant (*p* = 0.31). From the annual average flow rate of both rivers and the concentration difference between their plumes and Atlantic surface water, we estimate a net daily discharge in the order of 10^18^ INP_−15_ for the Amazon and 10^16^ INP_−15_ for the Tocantins.

A substantial fraction of INPs found in river water pass through a 0.22 µm filter [5–8]. In a limnological context, these small INPs would be referred to as dissolved organic matter (DOM). The quality of DOM in rivers clearly depends on land cover in the watershed area [26], which might explain some of the difference in INPs between the Tocantins and Amazon. The 10^1^ and 10^2^ INP_−15_ ml^−1^ in the Tocantins and the Amazon rivers, respectively, were three and two orders of magnitude lower than what has been reported from the lower reaches of the Mississippi River [7], the largest river in North America. Interestingly, the difference in INP_−15_ on decaying leaves from tropical and temperate regions is of the same direction and order of magnitude [10]. Therefore, despite an about tenfold smaller discharge, the Mississippi River might still transfer an order of magnitude more INPs from land to ocean than does the Amazon. Mississippi freshwater influences substantial areas in the northern Gulf of Mexico [27], where discharged INPs could become aerosolized by wave-breaking and bubble-bursting [28,29], as are INPs of marine origin in the Gulf of Mexico [30], perhaps with atmospheric implications. For example, at their coastal observatory in Yucatan, Ladino *et al*. [31] found an enhanced atmospheric concentration of INP_−15_ in marine air masses arriving with two cold fronts from northerly directions. Also in other regions, shelf waters enriched with INPs from large rivers could be an atmospherically relevant source of INPs to marine air masses. Furthermore, the origin of highly active INPs in the atmosphere above the North Pole has been traced back to the region of shallow waters over the Russian continental shelf [32]. Unfortunately, the INP concentration in Siberian rivers feeding these shelf waters is still unknown, but the analyses by Schnell & Vali [10,11] on decaying leaves from different climatic zones suggest even higher INP concentrations in the Siberian rivers than in rivers in temperate regions. In addition, thawing permafrost in Siberian river catchments probably adds further INPs that are discharged into shelf waters of the Arctic Ocean [33].

### 3.2. River water and the atmosphere can have different sources of INPs

The concentration of INPs was different between the two river plumes, as was the pattern of heat sensitivity in these INPs, although other parameters known to drive INP concentration—such as turbidity [8] and humic-like substances [34], of which fluorescence is a measure—were similar in both river plumes (table 1).

Typically, heat tolerance of INPs decreases markedly with increasing activation temperature, whether in soil [18,35], air [36–38], precipitation [37,39] or seawater [40,41]. Therefore, in the following we focus on a temperature window from −13 to −15°C and average all measurements made in 0.5°C steps within this window.

In the Tocantins water, 39% of all INPs were heat sensitive and only 25% were heat tolerant (table 2). In the Amazon water, few INPs were heat sensitive but 66% were heat tolerant. This fraction is nearly four times as large as the heat-tolerant fraction of INPs in airborne particles above the middle of the Amazon rainforest (18%, s.d. = 15%; derived from values in fig. 6 in [38]). An explanation for this discrepancy could be that INPs in the air above dense canopies and INPs in water below them are from separate and contrasting sources. Seven-day back trajectories indicate that air masses above the canopy had arrived from the Atlantic and crossed the coastline near the mouths of the Amazon and Tocantins rivers before reaching the air-sampling location about 1200 km further inland [38]. If the air masses contained INPs from the Amazon River plume, their signature in terms of heat sensitivity could have been unrecognizably diluted by further INPs taken up during the 1200 km journey westward over the Amazon rainforest.

**Table 2.** Fraction of INPs active between −13 and −15°C by category of heat sensitivity. The fraction of INPs lost in the 60°C treatment is termed heat sensitive; the fraction tolerating 60°C but deactivated by 95°C is termed moderately heat tolerant; and the fraction still active after both treatments is called heat tolerant. Mean values are for five measurement points on three and two samples from the Amazon and Tocantins river plumes, respectively (s.d. = standard deviation).

category of INPs	Amazon		Tocantins	
	mean	s.d.	mean	s.d.
heat sensitive	0.06	0.16	0.39	0.14
moderately heat tolerant	0.29	0.14	0.36	0.05
heat tolerant	0.66	0.18	0.25	0.13

During the rainy season, when samples were taken, much of the DOM in river water probably originates from compounds washed from litter and soil into the river [42], whereas aerosol particles above the canopy more likely are emitted from the canopy itself. When raindrops impinge on leaves and branches, they generate aerosols containing microorganisms from these surfaces. Driving mechanisms can involve bubble-bursting at the surface of water films by which tiny droplets (10–100 µm) and particles are aerosolized [43]. Other possibilities of aerosol generation by rain impact on canopies include micro-splashing [44]. Droplets thus produced can be smaller than 50 µm [45] and get lofted above a canopy [46], where they evaporate in the drier air [47] and enrich it with the INPs [48,49] that were initially contained in the splash droplets. Whatever the mechanism, the most likely source of INPs aerosolized by raindrops impinging on the canopy would be ice-nucleation active epiphytic microorganisms, most of which are heat sensitive and only some are moderately heat tolerant [20]. Heat-sensitive INPs washed by rain from canopies will mix on the ground with a probably much larger pool of INPs that themselves are very unlikely to be transported through and above the canopy of the tropical rainforest. At night, the air below the canopy is persistently decoupled from that above it [50,51]. During the daytime, larger turbulent structures may occasionally penetrate the dense foliage of the canopy and result in an occasional exchange of air and particles. However, observations of fluorescent biological aerosol particles above and below a rainforest canopy in Borneo have suggested only a weak coupling of larger aerosol particles between these layers [52]. Known heat-tolerant INPs that could be washed from litter and soil into the river are mineral particles [53], lignin [54] and other heat-stable organics [9,36], such as macromolecules of pollen [55]. A much stronger source of such heat-tolerant INPs below the canopy in the Amazon compared with the Tocantins watershed would explain (i) the greater abundance of INPs in the Amazon, (ii) the larger fraction of heat-tolerant INPs in the Amazon compared with the Tocantins River plume, and (iii) the difference in heat-tolerant fraction between INPs in the river plume and INPs in the air above the Amazon rainforest.

## 4. Conclusion

To conclude, land-use change probably affects the abundance and composition of INPs washed into watercourses. Although INPs might be produced at higher rates where rainforests are less degraded, the dense canopies of intact rainforests probably obstruct the stronger sources from emitting to the atmosphere. Therefore, the difference in INP concentration and heat-tolerance in the two investigated river plumes is unlikely to translate to similar differences in INPs emitted directly from their watersheds to the atmosphere above. Nevertheless, INPs discharged by the Amazon River into the Atlantic significantly increase the INP concentration in water above the continental shelf and could affect the INP concentration and composition in marine air masses. Although the Amazon is the largest river in terms of discharge, smaller rivers in temperate or cold regions might transfer even more INPs to shelf waters, because decaying leaves are a stronger source of INPs in colder climates than in a tropical climate. Variations in atmospheric INP concentration reported from the Gulf of Mexico and the Arctic Ocean lend initial support to the idea that INPs transported by large rivers from land to shelf waters could have a discernible impact on the atmospheric INP concentration in marine air masses.

## Data accessibility

The data are provided in electronic supplementary material [56].
